# Hydrogen Accumulation and Distribution in Pipeline Steel in Intensified Corrosion Conditions

**DOI:** 10.3390/ma12091409

**Published:** 2019-04-30

**Authors:** Anatolii I. Titov, Aleksandr V. Lun-Fu, Aleksandr V. Gayvaronskiy, Mikhail A. Bubenchikov, Aleksei M. Bubenchikov, Andrey M. Lider, Maxim S. Syrtanov, Viktor N. Kudiiarov

**Affiliations:** 1 PAO ‘Gazprom Transgaz Tomsk’, 9, Frunze Ave., 634029 Tomsk, Russia; office@gtt.gazprom.ru (A.I.T.); office@gtt.gazprom.ru (A.V.L.-F.); 2 OOO ‘Gazprom Transgaz Ukhta’, 10/1, Naberezhnaya Gazovikov, 169300 Ukhta, Komi Republic; sgp@sgp.gazprom.ru; 3 National Research Tomsk State University, 36, Lenin Ave., 634050 Tomsk, Russia; Aleksy121@mail.ru; 4 National Research Tomsk Polytechnic University, 30, Lenin Ave., 634050 Tomsk, Russia; lider@tpu.ru (A.M.L.); maxim-syrtanov@mail.ru (M.S.S.); viktor.kudiiarov@gmail.com (V.N.K.)

**Keywords:** trunk gas pipelines, hydrogen embrittlement, hydrogen adsorption, pipeline inner surface, cathodic protection current, hydrogen distribution

## Abstract

Hydrogen accumulation and distribution in pipeline steel under conditions of enhanced corrosion has been studied. The XRD analysis, optical spectrometry and uniaxial tension tests reveal that the corrosion environment affects the parameters of the inner and outer surface of the steel pipeline as well as the steel pipeline bulk. The steel surface becomes saturated with hydrogen released as a reaction product during insignificant methane dissociation. Measurements of the adsorbed hydrogen concentration throughout the steel pipe bulk were carried out. The pendulum impact testing of Charpy specimens was performed at room temperature in compliance with national standards. The mechanical properties of the steel specimens were found to be considerably lower, and analogous to the properties values caused by hydrogen embrittlement.

## 1. Introduction

Metal corrosion is currently one of the most important issues of oil and gas transportation [1]. Cumulative corrosion damage in the oil and gas industry is estimated to be more than $1 billion annually [2]. At the same time, revenue losses due to repair operations exceed $10 billion annually [3]. The United States Department of Transportation reported one of the major causes of pipeline failure to be stress corrosion cracking (SCC) [4]. In accordance with the pipeline classification (by soil type and structure, pressure, temperature, and cathodic protection) accepted by the expert community, SCC is caused by different mechanisms [5]. Propagation of stress corrosion cracking in a soil with pH higher than 9 is induced by cathodic protection currents and the coating condition, usually SCC occurs in carbonate-based electrolytes. The latter represent carbonic acid (H_2_CO_3_) which dissociates in water and forms -CO_3_ and -HCO_3_ which also appear in soil due to dissolution of carbon dioxide (CO_2_) [6]. This SCC mechanism includes intercrystalline fracture caused by the changes in chemical properties along the grain boundaries. In this case, primary dissolution and decrease in cohesive energy of the grain boundary inclusions are observed. This SCC mechanism occurs in Australia, Argentina, Iran, Saudi Arabia, the United States, and also in regions of Central Asia and Kazakhstan [7,8,9]. In Russia this mechanism is not observed.

Propagation of stress corrosion cracking in a soil with pH varying from 6–8 is caused by the mechanism of transcrystalline fracture that propagates through the grain, regardless of the grain boundary. Most of the theories explain this SCC mechanism by the synergetic effect caused by hydrogen dissolution in steel, corrosive medium, strain and adsorption of other substances on the pipeline inner surface. This results in reduction in the atomic binding energy of the crystal lattice. This SCC mechanism is observed in Canada, Italy and some regions of Russia and other countries [7,8,9].

According to [10,11], propagation of stress corrosion cracking in a soil with pH lower than 6 occurs due to the mechanism of hydrogen permeation in the pipe steel and its hydrogen embrittlement. Saturation of the steel pipe with hydrogen is possible in acid soil due to the free migration or electrophoretic mobility of H ions from corrosive environment to the pipe metal provided by the excessively intensive cathodic protection [12]. This SCC mechanism occurs mostly in China and Russia [13].

Atomic hydrogen absorbed into steel and diffused in it by the interstitial mechanism, is pressed from all sides of the crystal lattice except for defects (dislocations, vacancies, grain boundaries), and tends to be displaced therein. With the increase in the hydrogen amount by hundreds of times, its molecularization occurs, thereby creating the local pressure greater than 100 atmospheres [14,15,16]. Moreover, when hydrogen reacts with some of alloying elements, it forms hydrides that bring these elements out of the functional state of substitutional or interstitial solutions [14,15,16]. Hydride, carbide and hydride segregations, which form in dislocations or along the grain boundaries, lower their mobility and cohesive energy resulting in formation of coalescences with defects [14,15,16]. In addition, hydrogen causes reduction in the material hardness changing, the surface energy during the simple interpolation, i.e., the Rehbinder effect [17]. At the macro-scale level, hydrogen modifies the elastoplastic properties of steel and facilitates both reduction in its plasticity via hydrogen embrittlement, and localized plasticization of crack nuclei; in its turn, the localized plasticization facilitates cracks growth [7].

Hydrogen embrittlement is a very serious problem widely discussed by researchers worldwide. Researchers consider hydrogen embrittlement in relation to development of hydrogen energy technologies and pipeline transport of hydrogen rather than natural gas [18,19]. Hydrogen permeates a steel pipe not only from the outside but also from the inside due to dissociation of hydrocarbons (methane). This is because of either chemisorption of methane, the main gas component [20], on the steel surface and its transformation on the inner, slightly rusted surface at evaporation or CO_2_ conversion of methane [21]. Since VIII group metals Fe, Ni, Co can catalyze dehydrogenation reaction of methane [22], it is possible for hydrogen to adsorb on the inner surface as a reaction product. The rate of chemical conversion of methane is not zero even at 100 °C, although in industrial conditions it usually occurs at the temperature above 700 °C. This is due to the presence of molecules having higher kinetic energy as a result of the velocity distribution of gas molecules [23].

It is worth noting that when the products of the dehydrogenation reaction of methane are withdrawn from the reaction site which absorbs hydrogen atoms, the chemical equilibrium shifts to the right and, therefore, adsorption is the limiting factor of the inverse reaction [23]. 

### Methane Dissociation

The degree of dissociation of methane (CH_4_) molecules is determined by their chemisorption on Ni catalyst. The Gibbs free energy (Table 1) of CH_4_ decomposition reaction is indicated as:

H_4_ ↔ C + 2H_2_ (1)

The change in the Gibbs free energy is Δ*G* = Δ*H* − *T*Δ*S*, where *H* is the enthalpy, *T* is the absolute temperature, *S* is the entropy. This change describes the permissible process of the chemical reaction [23]. Thus, under the given thermodynamic conditions, the reaction occurs at ΔG < 0 and does not occur at ΔG > 0. As for the decomposition Reaction (1), ΔH < 0, ΔS < 0 and ΔG < 0 at T < 929 K. Hence, methane dissociation occurs due to the enthalpy of formation only and is typical for low temperatures. The literature presents disaggregated data [25] on methane dissociation on iron oxides and hydroxides. Nevertheless, it can be measured by the upper level of its efficiency using the data on methane dissociation on Ni catalysts because they are more efficient than Fe [26]. According to [27], the reaction equilibrium constant of methane dissociation at T = 373 K is written as

(2) $$K_{p}^{1}=0.606\times 10^{-7}$$

This indicates the absolutely permissible methane dissociation on the inner surface of the pipeline under the conditions approaching to normal.

As for other permissible transformations of methane, the reactions of combination of methane and water vapour, carbon dioxide, and oxygen can occur by the following mechanisms [28,29]:

CH_4_ + H_2_O ↔ CO + 3H_2_ (3)

CH_4_ + 2H_2_O ↔ CO_2_ + 4H_2_ (4)

CH_4_ + CO_2_ ↔ 2CO + 2H_2_ (5)

CH_4_ + O_2_ ↔ C + 2H_2_O (6)

2CH_4_ + O_2_↔ 2CO + 4H_2_ (7)

CH_4_ + 2O_2_ ↔ CO_2_ + 2H_2_O (8)

Reactions (2) and (3) are steam methane reforming; Reaction (4) is CO_2_ conversion; Reactions (5) and (6) are partial oxidation, and Reaction (7) is complete oxidation or burning of methane. Reactions (2) and (3) describe the same process, provided that Reaction (3) describes further CO_2_ conversion. For Reactions (5–7) oxygen is required, however, its content in natural gas is very low [30]. 

The reactions of methane conversion (2–4) are usually performed at the temperature over 400 °C and 1–4 MPa pressure, in the presence of catalysts [31,32]. This is because of their high activation barrier due to relatively high energy of methane dissociation or its C–H chemical bonds, such as the first 435 kJ/mole bond, the second and third bonds 444 kJ/mole each, and the fourth 335 kJ/mole bond [26]. According to [31,32], Pt, Pd, Co, Ni, Fe and Cu and their oxides are more efficient catalysts for such reactions. 

It is assumed that steam methane reforming consists of two stages:

CH_4_ + H_2_O ↔ 3H_2_ + CO (−206 kJ) (9)

CO + H_2_O ↔ H_2_ + CO_2_ (+42 kJ) (10)

The first stage is strongly endothermal and critical to steam methane reforming. Both stages are reversible reactions. 

Measurements of the Gibbs free energy for steam methane reforming demonstrate that Δ*G* > 0 at *T* = 373 K. According to [29], at this temperature, the reaction equilibrium constant for steam methane reforming is

(11) $$K_{p}^{1}=0.269\times 10^{-19}$$

Therefore, hydrogen formation on the inner surface and in the depth of the pipe can be explained by methane chemisorption. This is proven by the data about carbon forming on the inner pipe surface [33].

In compliance with the national standards [30], the concentration of water vapour in the transported natural gas composition should not exceed 30 mg/m^3^. Hydrostatic testing of gas pipelines demonstrates the water remains on the inner surface of the pipeline in the amount of 30 tons per 100 km owing to its non-zero wettability and roughness [13]. Despite the traditional purification of natural gas from CO_2_ with the view to prevent crystallohydrate formation, it may contain up to 30 g/m^3^ CO_2_ [30].

The possibility of low-temperature methane transformations is supported by the nature. Thus, iron is used as a catalyst not only in chemical industry technologies. Obligate methanotrophs are methane loving microorganisms which receive energy and raw material for cellular structures necessary for life from methane [34] and oxidize methane such that

CH_4_ + O_2_ + NAD(P)H + H^+^ ↔ CH_3_OH + NAD(P)^+^ + H_2_O (12)

Methanotrophs use methane monooxygenase (MMO), an enzyme that exists in two forms, namely the soluble form (sMMO) and the particulate form (pMMO). The active site in sMMO contains an iron atom, whereas the active site in pMMO utilizes copper [34]. There are several thousand species of methanotrophs that effectively develop at the temperatures ranging from 10–70 °C [34]. Some species of methanotrophs live deep underwater in the arctic region. Therefore thermodynamic conditions inside gas pipelines with the temperature range of 5–15 °C and 25–70 atm pressure and iron oxides and hydroxides on the inner surface are comparable to methanotrophs thermodynamics. Most of methanotrophs are aerobic and cannot live inside gas pipelines because of the insufficient oxygen percentage of 0.001% in transport gas. There are, however, anaerobic methanotrophs which are currently being investigated [34]. Consequently, the possibility of reaction of methane decomposition at low temperatures is confirmed by measurements of the Gibbs free energy and metabolic capabilities of methanotrophs.

The goal of this research is to prove the possibility of hydrogenation of the inner surface of gas pipelines caused by dissociation of hydrocarbons. A gas reactor is designed to simulate the operating conditions for the real gas pipeline. Hydrogen accumulation and distribution in steel specimens are studied in the proposed gas reactor under the conditions of the accelerated corrosion test.

## 2. Materials and Methods

On the territory of the Russian Federation for the construction of trunk gas pipelines, structural low-alloy steels of grades 09G2S and X70 are commonly used, since their characteristics allow for operating under the pressure within the wide temperature range (−70, +450 C), being durable and resistant to dynamic loads. Therefore, a pipe made of 09G2S steel was chosen as a sample for the experiment. The production technology of this steel does not imply extensive use of hydrogen at the stage of dispersal, therefore its initial content in steel after smelting remains negligible. By its composition, steel 09G2S most closely relates to international analogues—steel grades A 516-55, A 516-60, A 516-65, A 561 Gr70, produced in the USA. The group of these steels demonstrates resistance to sulfide stress corrosion cracking (SSCC) and is recommended for the use with acid gases. At the same time, there is sufficient data on the susceptibility of these steels to stress corrosion cracking [6,7,8,9], which in some cases is also explained by hydrogen damage. Hydrogen-induced cracking (HIC) test results demonstrate that high-strength steels are most susceptible to it, and in the series of X52, X60, X70, and 100XF steels, the latter is most vulnerable. However, X70 also proved to be sufficiently exposed to HIC, which makes it possible to speak of the possibility of its corrosion stress cracking, which develops with the help of HIC. 

**Figure 1:**
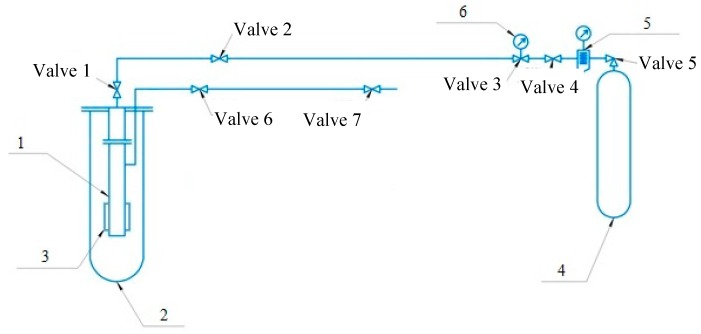
Simulation system for the inner surface hydrogenation in the intensified corrosion conditions: 1—reactor, 2—casing, 3—heater element, 4—methane gas canister, 5—pressure controller, 6—manometer.

The gas reactor supplying methane-based mixture was designed to simulate the corrosive environment matching the operating conditions for the real gas pipeline that enabled intensification of the studied processes increasing the temperature and humidity. The simulation system for hydrogenation of the pipe inner surface is illustrated in Figure 1.

Table 2. summarizes the gas mixture components supplied to the gas reactor.

The proposed gas reactor is a closed steel pipe prepared for operating under pressure. The steel pipe has two valves. Valve 1 supplies gas in the reactor, whereas valve 6 serves for gas sampling. Reactor 1 has a removable pipe section just above heater element 3 which is withdrawn for examination from the reactor after the experiment. Steel specimens (templates) are then cut from the removable pipe section and analyzed.

The gas mixture is supplied to the reactor under 40 atm pressure which is measured with manometer 5. The composition of the gas mixture is given in Table 2. The installation was designed so that it would be possible to conduct studies of steel samples subjected to aggressive impact both from the outside and from the inside. However, the goal of this study was to investigate the concentration and distribution of hydrogen adsorbed in the process of methane chemisorption inside the pipe, and therefore there was no aggressive action outside.

The 3 kW nickel chromium clamp heater is connected to the temperature controller and two thermocouples mounted inside the reactor. The thermocouples are accurate to within 0.1 °C, with 1° accuracy of the controller, and 100 °C heating stop temperature. The heater element allows the corrosion tests to be intensified at various temperatures, because, in accordance with the main statements of chemical kinetics, it affects the rate of chemical reactions.

**Figure 2:**
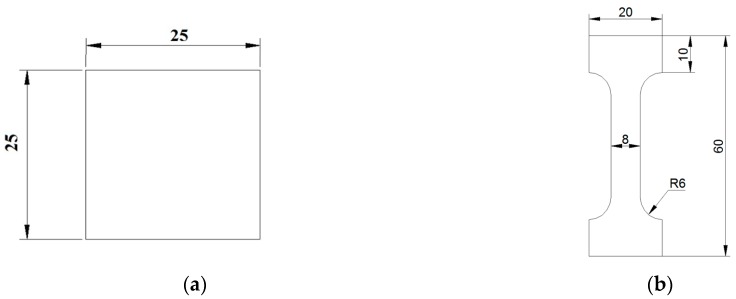
Specimens dimensions in millimeters: (**a**)—for elemental analysis, (**b**)—for uniaxial tension tests.

In order to conduct the uniaxial tension tests and determine the elemental composition of the pipe, the electro discharge machining was used to cut off specimens from the pipe center parallel and normal to the axial direction. The X-ray diffraction (XRD) analysis was carried out on templates 2 mm thick cut off from the pipe at various depth. For the uniaxial tension tests, the dog bone specimens were cut off in compliance with the ISO 6892-84 [29,30,31,32], as presented in Figure 2. 

The steel specimens were studied in two states, before and after 24-h exposure to the humid and heating environment. The templates and dog bone specimens were obtained from the treated specimens for further investigations.

The phase composition of the templates was investigated on the XRD-7000S X-ray diffractometer (Shimadzu, Japan), and the obtained diffraction patterns were analyzed. Measurements were conducted using copper radiation (*K*_α1_, *K*_α2_). The operating parameters for the XRD-7000S included 10–90° scan range; 0.0143° step angle; 2.149 s exposure time. The XRD patterns were recorded on the OneSight wide-range array detector with 1280 channels (Shimadzu, Japan).

The qualitative and quantitative elemental analysis of the treated steel specimens was performed on the GD-Profiler 2 spectrometer (Horiba Scientific, Japan) which combines glow discharge powered by the radio frequency source with the optical emission spectrometer (RF-GD-OES).

The test machine Com-Ten Industries DFM-5000 was used in this experiment for tensile testing under the dead load. Metal parts with curved square section 5 × 5 mm^2^ were cut from pipe and then were mechanically grinded. Then flat specimens with square section 4.5 × 4.5 mm^2^ were tested on the impact pendulum Instron 450MPX impact tester (UK) at room temperature. The impact testing was carried out in compliance with national standards.

## 3. Results and Discussion

### 3.1. XRD Analysis

**Figure 3:**
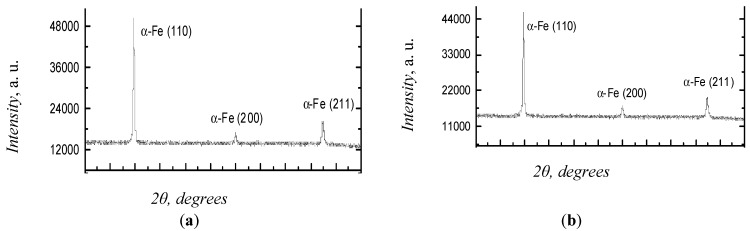
XRD patterns of the steel specimens: (**a**)—before exposure to; (**b**)—after exposure.

The XRD analysis of the specimens was performed before and after 24-h exposure to humid and heating environment. Five specimens were used for each test. The diffraction patterns obtained for these specimens are given in Figure 3.

The analysis of the diffraction patterns demonstrates that all the specimens contain only the α-Fe phase with the body-centered cubic (BCC) lattice. Table 3 presents the lattice parameters, the average grain size estimated by the XRD data-based calculations of the size of the coherent scattering region, and the microstress values.

As can be seen from Table 3, long-term exposure of steel specimens to the corrosive environment leads to the microstress relaxation without any changes in the phase composition and lattice parameters. 

### 3.2. Elemental Composition

The elemental composition of the steel templates was examined both on the inner and outer surfaces of the pipeline. Table 4 summarizes the elemental composition detected by the RF-GD-OES for the outer and inner surfaces of the untreated specimens. The weight content of the chemical elements is presented as a result of ten measurements. The relative uncertainty of measurements is about 1%. 

The elemental analysis reveals that both surfaces of the untreated specimens are similar. The relative difference in the weight content is 1%. The elemental composition matches AISI 1513 carbon steel grade.

The RHEN 602 gas analyzer (LECO, USA) is used to measure the absolute hydrogen concentration in the untreated steel specimens cut off from the inner and outer surfaces and from the bulk. The obtained results are presented in Table 5. The hydrogen concentration values were obtained after three experiments. In calculating the standard deviation of the mean, the confidence figure was assumed to be 0.95; the Student’s coefficient was 4.3.

Within the measurement uncertainties, the hydrogen concentration in the specimens before the treatment is similar in all the pipe sections. Table 6 presents the RF-GD-OES data for the elementary composition of the outer and inner surfaces of the treated specimens. The weight content of the chemical elements is presented as a result of ten measurements. The relative uncertainty of measurements is about 1%. 

According to the elemental analysis, the outer and inner surfaces of the steel specimens exposed to the corrosive environment are similar. The relative difference in the weight content ranges within 1%. The elemental composition matches AISI 1513 carbon steel grade.

The results of the gas analysis of the absolute hydrogen concentration in the treated steel specimens cut off from the inner and outer surfaces and from the bulk are presented in Table 7. The hydrogen concentration values are obtained after three experiments. In calculating the standard deviation of the mean, the confidence figure was assumed to be 0.95; the Student’s coefficient was 4.3.

The gradient hydrogen distribution is observed in steel specimens after their treatment in the gas environment, humid and heated. On the inner surface and in the bulk of the material the H concentration is 4 times higher than on the outer surface. The decrease in the hydrogen content on the outer surface of the pipe after treatment may be due both to the diffusion of hydrogen into the pipe bulk and to the partial desorption of hydrogen as a result of heating.

The H concentration on the inner surface in the initial steel state is 0.00065 wt.%, whereas after the gas treatment it is 0.00086 wt.% i.e., hydrogen permeates the steel when subjected to the gas treatment during heating.

### 3.3. Uniaxial Tension Tests

**Figure 4:**
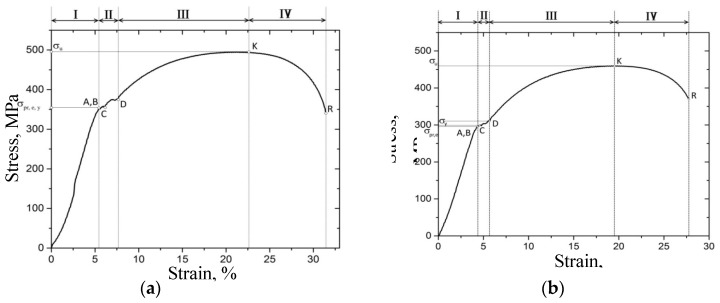
The stress-strain curves: (**a**)—before treatment, (**b**)—after treatment.

The uniaxial tension tests were performed at room temperature. The dog bone specimens were cut off from the pipe steel in its initial state and after treatment in the corrosive environment. Such cases are presented in Figure 4.

Table 8 presents the main parameters of steel specimens before and after treatment.

According to Table 8, the effect from the humid gas environment on the heated specimen results in significant degradation of its mechanical properties. Thus, the values of the proportional limit strength, elastic limit strength and the yield strength are reduced by 20%; tensile strength decreases by 7% and the maximum percent elongation decreases by 18%.

### 3.4. Pendulum Impact Testing

The pendulum impact testing of Charpy specimens was carried out at room temperature in accordance with the national standards. The impact testing results are summarized in Table 9.

The impact tests reveal that the resilience of the untreated and treated specimens is 355 and 345 J/cm^2^ respectively. These values are averaged by three dimensions for each specimen. There is no serious change in both states of specimens due to the low H concentration which does not exceed the solubility limit of hydrogen in steel. The impact of aggressive environment and the accumulation of hydrogen influence on the behavior of the material in the process of uniaxial stretching. However, these factors did not affect the behavior of the material when tested for impact strength. The reasons for this will be investigated further.

## 4. Conclusions

The effect the corrosive environment produces on the structure, elemental composition, mechanical properties and the impact toughness of the pipe steel have been studied. The XRD analysis, optical spectrometry and uniaxial tension tests revealed that the corrosion environment affects the parameters of the inner and outer surfaces of the steel specimens as well as the steel pipeline bulk.

In the initial state, on the inner surface of the specimens H content was 0.00062 wt.%, whereas after exposure to the corrosive heating environment it reached 0.00086 wt.%. That indicated hydrogen permeation in the steel during the experiment. Hydrogen accumulation resulted in steel embrittlement proven by the mechanical tests.

## Figures and Tables

**Table 1:** Standard values of the Gibbs free energy, enthalpy and entropy of formation of substances involved in Reaction (1) [24].

Substances	CH_4_	C	H_2_
Standard Gibbs free energy, Δ*G*^0^, kJ/mole	50	0	0
Standard enthalpy of formation, Δ*H*^0^, kJ/mole	74.6	0	0
Standard entropy of formation, Δ*S*^0^, kJ/mole	186.19	5.7	130.5

**Table 2:** Percentage of gas mixture components.

Substance	CH_4_	C_2_H_6_	C_3_H_8_	C_4_H_10_	CO_2_	H_2_S
Weight content, %	97	1.5	0.5	10^−2^	10^−3^	10^−5^

**Table 3:** XRD data.

Treatment	Phases	Phase Composition, vol.%	Lattice Parameters	Microstress
Before	BCC α-Fe phase	100	*a* = 2.8697	0.001065
After	BCC α-Fe phase	100	*a* = 2.8692	0.000425

**Table 4:** Elemental composition of the outer and inner surfaces in the untreated specimens.

Chemical Elements	Weight Content, % (Outer Surface)	Weight Content, % (Inner Surface)
C	0.078	0.072
Si	0.321	0.352
Mn	1.331	1.266
S	0.002	0.003
Fe	98.268	98.307

**Table 5:** H content in the untreated steel specimens.

Weight Content, wt.%	Outer Surface	Bulk	Inner Surface
H content	0.00069	0.00067	0.00065
Direct measurement error	0.00005	0.00004	0.00003

**Table 6:** Elemental composition of the outer and inner surfaces in the treated specimens.

Chemical Elements	Weight Content, % (Outer Surface)	Weight Content, % (Inner Surface)
C	0.075	0.071
Si	0.318	0.339
Mn	1.315	1.331
S	0.004	0.004
Fe	98.288	98.255

**Table 7:** H content in the treated steel specimens.

Weight Content, wt.%	Outer Surface	Bulk	Inner Surface
H content	0.00020	0.00083	0.00086
Direct measurement error	0.00005	0.00006	0.00004

**Table 8:** Main parameters of steel specimens in different states.

	Before Treatment	After Treatment
Proportional limit strength ± 20, MPa	352	296
Elastic limit strength ± 20, MPa	352	296
Yield strength ± 20, MPa	352	300
Tensile strength ± 20, MPa	494	462
Maximum percent elongation ± 0.1, %	32	27

**Table 9:** Fracture toughness of steel specimens in different states.

Resilience KCV, J/cm^2^	Initial State	State after Treatment
KCV ± 20	355	345
